# Evaluation of strategies for improving the transgene expression in an oleaginous microalga *Scenedesmus acutus*

**DOI:** 10.1186/s12896-018-0497-z

**Published:** 2019-01-10

**Authors:** Anongpat Suttangkakul, Anchalee Sirikhachornkit, Piyada Juntawong, Wilasinee Puangtame, Thitikorn Chomtong, Suchada Srifa, Sukhita Sathitnaitham, Wasawat Dumrongthawatchai, Kanidtha Jariyachawalid, Supachai Vuttipongchaikij

**Affiliations:** 1 Special Research Unit in Microalgal Molecular Genetics and Functional Genomics (MMGFG), Department of Genetics, Faculty of Science, Kasetsart University, 50 Ngam Wong Wan road, Chatuchak, Bangkok, 10900 Thailand; 2 Center of Advanced studies for Tropical Natural Resources, Kasetsart University, 50 Ngam Wong Wan road, Chatuchak, Bangkok, 10900 Thailand; 3 PTT Research and Technology Institute, PTT Public Company Limited, Ayuthaya, 13170 Thailand

**Keywords:** *Agrobacterium*-mediated transformation, Biofuel, Fluorescence reporter, Introns, Lipid, Microalgae, Mutagenesis, Transcript-fusion

## Abstract

**Background:**

Genetic transformation of microalgae has been hampered by inefficient transgene expression, limiting the progress of microalgal biotechnology. Many vector tools and strategies have been developed in recent years to improve transgene expression in the model microalga *Chlamydomonas*, but these were hardly applied to other microalgae. In this work, naturally-isolated oleaginous microalgae were accessed for genetic transformation, and various expression systems were evaluated in a selected microalga to circumvent inefficient transgene expression.

**Results:**

Initially, a strain of *Scenedesmus acutus* was selected from the oleaginous microalgal collection based on its highest transformation rate and transgene stability. This strain, which had very low or no GFP reporter expression, was first tested to improve transgene expression by using intron-containing constructs and the transcript fusion using *ble::E2A*. The intron-containing constructs yielded 2.5–7.5% of transformants with 2–4-fold fluorescence signals, while the majority of the transformants of the transcript fusion had the fluorescence signals up to 10-fold. Subsequently, three UV-induced *S. acutus* mutants were isolated with moderate increases in the level and frequency of transgene expression (2–3-fold and 10–12%, respectively). Finally, a transcript fusion system was developed using *psy* white mutants with an expression vector containing *PSY::E2A* for complementation and light selection. Transformants with green colonies were selected under light exposure, and the transgene expression was detected at protein levels. Although the improvement using *PSY::E2A* was only minor (1–2-fold increase and ~ 7% of transformants), this system provides an alternative selectable marker that is compatible with large-scale culture*.*

**Conclusions:**

Here, the overall improvement of transgene expression using the *Chlamydomonas* tools was moderate. The most effective tool so far is the transcript fusion using *ble::E2A* system. This work demonstrates that, so far, genetic engineering of non-model microalgae is still a challenging task. Further development of tools and strategies for transgene expression in microalgae are critically needed.

**Electronic supplementary material:**

The online version of this article (10.1186/s12896-018-0497-z) contains supplementary material, which is available to authorized users.

## Background

Oleaginous microalgae emerged as a promising renewable feedstock for biofuel production [1–3]. These microalgae can be cultivated using wastewater, seawater or simple medium and, under certain conditions such as nutrient deprivation or stresses, they can accumulate high lipid contents [4]. For decades, microalgae have been isolated and screened from natural sources for properties beneficial to biofuel production including rapid cell growth, high lipid yields, tolerance to stresses, ease of harvest and ability to secrete lipid [5]. To date, however, these microalgae are unable to produce biofuels at competitive costs compared to those of fossil fuels. Genetic improvement is a major step for making algal biofuels economically feasible [6, 7].

Genetic transformation has not been widely used in microalgae, partly because of difficulties in nuclear gene transformation, as reported in the model microalga *Chlamydomonas reinhardtii* and other species for low transformation efficiencies [8], inefficient transgene expression [9], transgene cleavages [9, 10], transgene instability [11] and highly control transcriptional silencing [12, 13]. Genome editing was also proved to be very difficult as the homology-directed repair (HDR) is extremely inefficient [14, 15] and CRISPR/Cas9 system was shown to be toxic for the algal cell [16]. The only exception so far is a marine microalga *Nannochloropsis*, which was reported for an efficient nuclear transformation and its feasibility for genome editing by either HDR [17] and CRISPR/Cas9 [18]. Initially, many attempts were made for improving transgene expression in *Chlamydomonas* by codon optimization [19] and screening large numbers of transformants to minimize transgene positional effects [20], but little improvement was achieved. Recent developments have demonstrated marked improvements by a number of approaches including mutant isolations for reduced transcriptional silencing [21] and the incorporation of introns in the expression constructs [22] and transcript fusion system [23]. However, most of these tools were developed for *Chlamydomonas,* and only a few have been applied in other microalgae [24].

Various oleaginous microalgae have been tested for genetic transformation, and, indeed, they suffer from inefficient transgene expression as *Chlamydomonas* had in the past [25, 26]. Notably, most of the work reported so far relied on the gene or enzymatic activity to represent the transgene expression, without confirmation at protein levels [24, 27–29]. This shortcoming is problematic as reliable detection at protein levels is the key indicator for establishing stable transgene expression, before evaluating the effects of transgenes such as phenotypes or compound production. Along the line of improvements in *Chlamydomonas*, many developed tools and strategies are now available, and it would be proven fruitful if these could be used for improving other oleaginous microalgae.

Here, ten oleaginous microalgae isolated from natural sources in Thailand were tested for genetic transformation. Initially, some of these microalgae were tested by electro-transformation, but very few or no transformant was obtained. In contrast, *Agrobacterium*-mediated transformation yielded much more transformants and was used to access the transformation efficiency of the ten microalgae. A strain of *S. acutus*, which has rapid growth and high lipid accumulation, was selected based on its high transformation rate and transgene stability. As expected, this strain has a low capability for transgene expression as indicated by very low levels of GFP reporter. This strain was then used for evaluating various expression tools, which were previously developed in *Chlamydomonas*, whether they could mitigate the low transgene expression problem. These included the use of constructs containing introns, constructs with transcript fusion and mutant isolation for reduced transcriptional gene silencing. Furthermore, a transgene expression system using novel selectable marker was developed by generating *psy* white mutants and complementation using *PSY* gene and light as the mean of selection. Transgene expression was analyzed for both expression levels and the frequency of expressors among the transformants.

## Results

### *Agrobacterium*-mediated transformation of wild-isolated oleaginous microalgal strains

Ten wild-isolated freshwater unicellular microalgae with high lipid accumulations were tested for *Agrobacterium*-mediated transformation using a method based on Kumar et al. [30]. Before transformation, axenic cultures of the ten strains were verified for hygromycin B sensitivity to estimate the selective concentrations (Additional file 1). After transformation using *Agrobacterium* harboring pCXSN-*GFP*, hygromycin B resistant colonies were obtained from six out of ten strains with transformation rates approximately 10–200 CFU per 10^6^ cells (Table 1, see also Additional file 2 for an example of selection plates). Some background colonies conferring spontaneous resistant to hygromycin B were observed from TISTR8511, 8555, 8519, 8540 and 8447, accounting for 5–9% of total colonies counted in transformation plates. Two *S. acutus* strains with the highest transformation rates, TISTR8540 and 8447*,* were tested for assessing the transgene stability. After 10–12 rounds of subcultures on the non-selective medium before transferring onto the hygromycin B selective medium, the growth of the TISTR8447 transformants was more consistent on the selective medium than those of TISTR8540 transformants (Additional file 3). This result suggests the higher stability of transgenes in TISTR8447. This *S. acutus* TISTR8447 was used for subsequent studies.

**Table 1:** Transformation rates of ten microalgal strains by *Agrobacterium*-mediated transformation

Species	Algal media	Concentration of hygromycin B (μg ml^− 1^)	Selection period (days)	Transformation rate (CFU per 10^6^ cells)	Background control (CFU per 10^6^ cells)
*Coelastrum* sp. (TISTR 8511)	TAP	10	15	0 (*n* = 44)	0
*S. acutus* (TISTR 8555)	TAP	30	15	20 ± 5.5 (*n* = 12)	1.5 ± 1.3
*S. acutus* (TISTR 8540)	TAP	50	10	72 ± 41 (*n* = 8)	3.7 ± 3.5
*S. acuminatus* (TISTR 8519)	TAP	50	10	31 ± 23 (*n* = 11)	2.7 ± 2.5
*S. acutus* (TISTR 8447)	TAP	30	15	217.5 ± 75 (*n* = 12)	19.5 ± 6.8
*C. humicola* (TISTR 8434)	TAP	20	15	10.8 ± 32 (*n* = 4)	0
*M. braunii* (TISTR 8429)	BG-11	10	15	0 (*n* = 8)	0
*A. falcatus* (TISTR 8557)	BG-11	10	15	0 (*n* = 12)	0
*T. cumbricus* (TISTR 8480)	TAP	50	18	9 ± 8.8 (*n* = 5)	0
*A. densus* (TISTR 8505)	BG-11	10	15	0 (*n* = 8)	0

### Evaluation of TISTR8447 as a platform for gene expression

TISTR8447 is a fast-growing strain, reaching its stationary phase within 5 days in TAP medium. It accumulates high lipid contents up to 3 ± 0.17 and 10 ± 1.04% dry weight in N-supplemented and N-deprived media, respectively, based on total lipid quantification using vanillin staining. The lipid content in this strain is somewhat in moderate levels for lipid production as compared to other potential feedstock microalgae [31]. Nile red staining also showed the abundance of lipid droplets in the cells (Additional file 4). TISTR8447 was further tested for transformation efficiency using four *Agrobacterium* strains: A41, EHA105, GV3101 and LBA4404. Despite no statistical difference among the *Agrobacterium* strains (*p >* 0.05), EHA105 and GV3101 appeared to provide the highest transformation rates (Additional file 5). The effect of Acetosyringone was tested, but no significant difference among the tested concentrations (0, 50, 100 and 200 μM) was observed (*p >* 0.05, data not shown), indicating that Acetosyringone is not required.

Transformed TISTR8447 were analyzed for the presence and expression of the transgene. Among 15 randomly selected potential-transformants subjected to either PCR or RT-PCR analysis, the *aphIV* gene (hygromycin B resistant gene) and its transcript was detected in 12 and 13 transformants, respectively (Additional file 6). This analysis indicates that most of the hygromycin B resistant colonies contained the transgene. However, after analyzing more than 60 transformants by confocal microscopy, little or no GFP signal was detected above the autofluorescence background (Additional file 6). RT-PCR of the *GFP* transcript and immunoblot analysis using anti-GFP antibody also failed to detect the presence of the transcript and protein (data not shown). These data demonstrate that TISTR8447 is a potential microalgal strain for biofuel production through its properties for growth, lipid accumulation and its ability to be efficiently transformed by *Agrobacterium*, but has a very low capacity for transgene expression.

### Transgene expression in TISTR8447 using *Chlamydomonas* introns

**Fig. 1:**
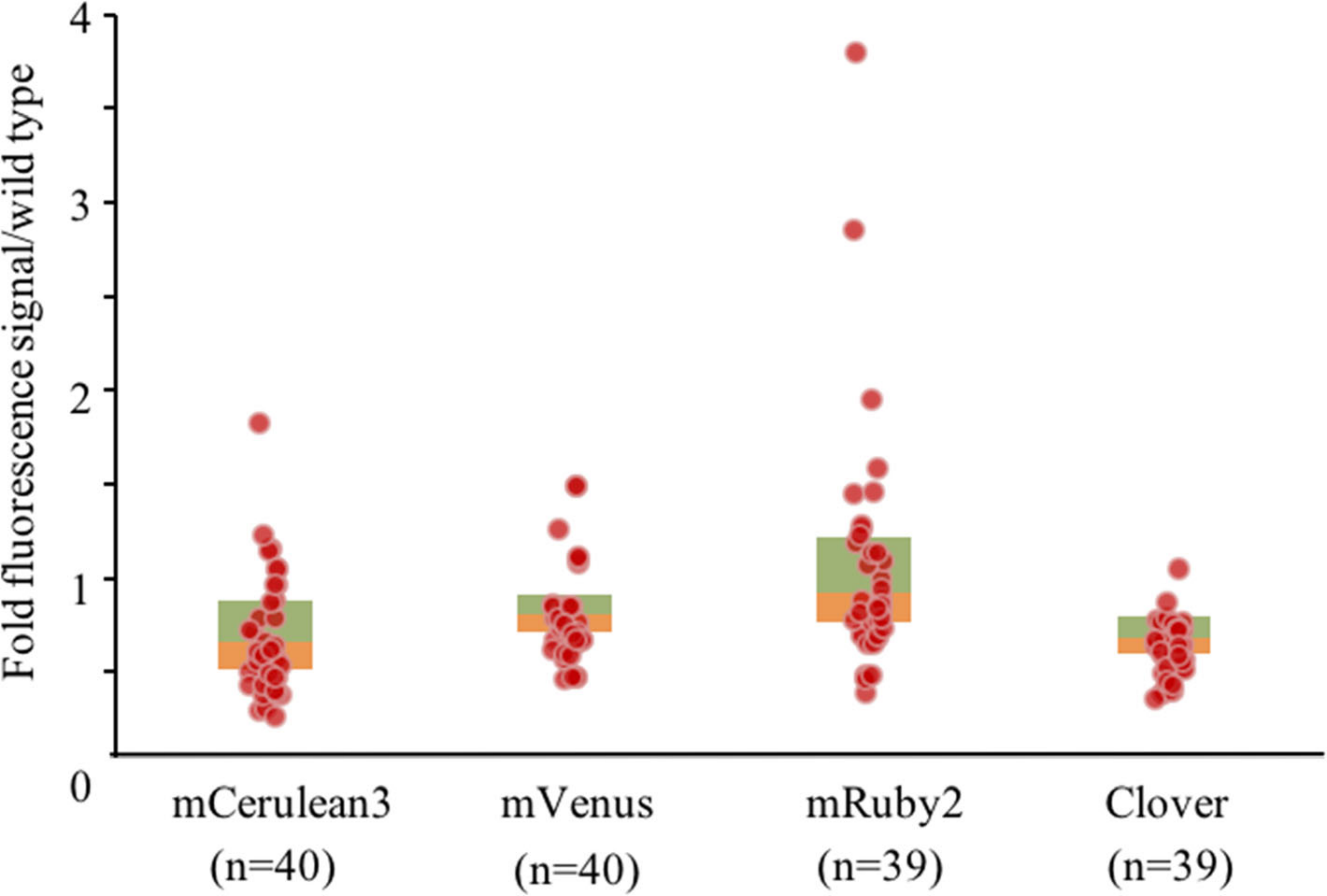
Analysis of TISTR8447 transformants expressing four fluorescence reporter genes harboring *Chlamydomonas* introns using fluorescence microplate reader. Data are presented as box plots overlaid with scatter plots for fold fluorescence reads over the wild type values. n indicates the number of transformants reads for each reporter

To test the effect of introns in transgene expression, four expression constructs with *Chlamydomonas* intron-carrying *mRuby2, mCerulean3, mVenus* and *Clover* reporters, which were previously shown to improve transgene expression in *Chlamydomonas* [22], were tested in the TISTR8447 through electro-transformation. Our electro-transformation rate for TISTR8447 was only 4.95 ± 5.18 CFU per 5 × 10^6^ cells (*n* = 20), and no background colony was observed when using paromomycin selection. Noting that electro-transformation of *S. obliquus* was previously reported as 494 ± 48 CFU per 10^6^ cells [25]. Fluorescence microplate analysis showed that most of the transformants had very low fluorescence signal ratios for the four reporters, similar to the levels observed in non-transformed controls (Fig. 1). Only a few showed 2–4-fold signal ratios indicating the expression of the reporter proteins above the background signals. The frequency of the expressers could be estimated 1–3 among 40 transformants (2.5–7.5%), which, nonetheless, is an improvement from the transformation using the pCXSN-*GFP*. This result indicates that the incorporation of *Chlamydomonas* introns can partly improve transgene expression in TISTR8447.

### Improving transgene expression in TISTR8447 using *ble::E2A* transcript fusion

**Fig. 2:**
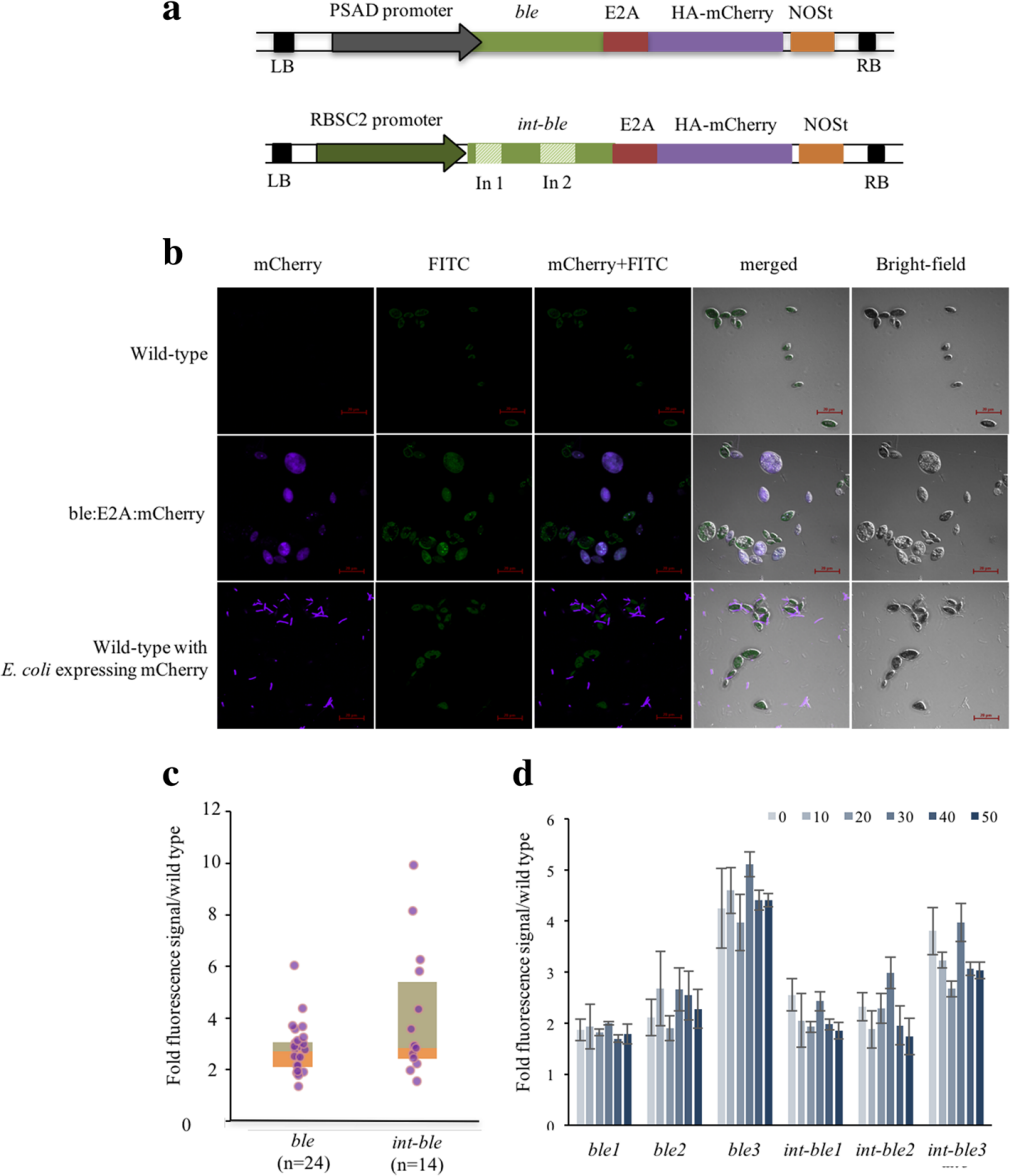
Analysis of TISTR8447 transformants expressing transcript fusions. **a** pCreZ-*ble::E2A::mCherry* (*ble*) and pCreZ-*intble::E2A::mCherry* (*int-ble*) vectors are illustrated. **b** TISTR8447 transformants analyzed by confocal microscopy showing mCherry signal and FITC channel-autofluorescence (scale bar = 20 μm). *E. coli* expressing *mCherry* was added to TISTR8447 wild type as a positive control. **c** mCherry signals of *ble* and *int-ble* vector transformants are presented as box plots overlaid with scatter plots. (n) indicates the number of transformants. **d** Fluorescence signals of low, medium and high mCherry expressors from each construct after subjected to media supplemented with 0–50 μg ml^− 1^ zeocin for 2 days. Error bars represent standard deviations (*n* = 3)

Previously, transcript fusion constructs using a self-cleavage 2A peptide from foot-and-mouth disease virus (FMDV) [32] with *ble* selectable marker [33] were shown to be a strategy of choices for highly efficient transgene expression in *Chlamydomonas* [23, 34–37]. To test whether this could improve transgene expression in TISTR8447, *ble::E2A* fusion vectors were constructed using with- or without-introns *ble* marker and *mCherry* reporter (pCreZ-*intble*::*E2A*-*mCherry* and pCreZ-*ble*::*E2A*-*mCherry*, Fig. 2 and Additional file 7). Transformants of these vectors were analyzed for the fluorescence reporter signal, and mCherry was readily observed under the confocal microscope (Fig. 2b). Fluorescence microplate analysis showed that most of the transformants from both constructs (19/24 or 79% for *ble* and 12/14 or 85% for *int-ble*) had more than a 2-fold increase of mCherry signal ratio to the wild type controls (Fig. 2c). The use of *RBSC2* promoter and introns in the *ble* gene (*int-ble*) showed higher signals compared to that using *PSAD* promoter and *ble* gene without introns (*ble*), though without significant difference by Kruskal-Wallis Test (*p >* 0.05). This result indicates that the *ble::E2A* transcript fusion system could provide an efficient transgene expression system for the TISTR8447 under selective pressure of zeocin.

Subsequently, the expression level of the *ble::E2A::mCherry* transcript fusion was tested whether it could be induced in a dose-dependent manner with elevated zeocin concentrations. Selected transformants with low, medium and high expression were cultured in at 10 μg ml^− 1^ zeocin, before transferring into the media supplemented with 0–50 μg ml^− 1^ zeocin for 2 days. However, no significant increase of the fluorescence signals following zeocin concentrations for both constructs was observed (Fig. 2d). At concentrations above 20 μg ml^− 1^ zeocin, the transformants grew poorly with low cell mass and became chlorosis. This observation demonstrates that increases in zeocin selective pressure could not provide a further improvement on transgene expression in the TISTR8447.

### Improving transgene expression in TISTR8447 by UV mutagenesis

**Fig. 3:**
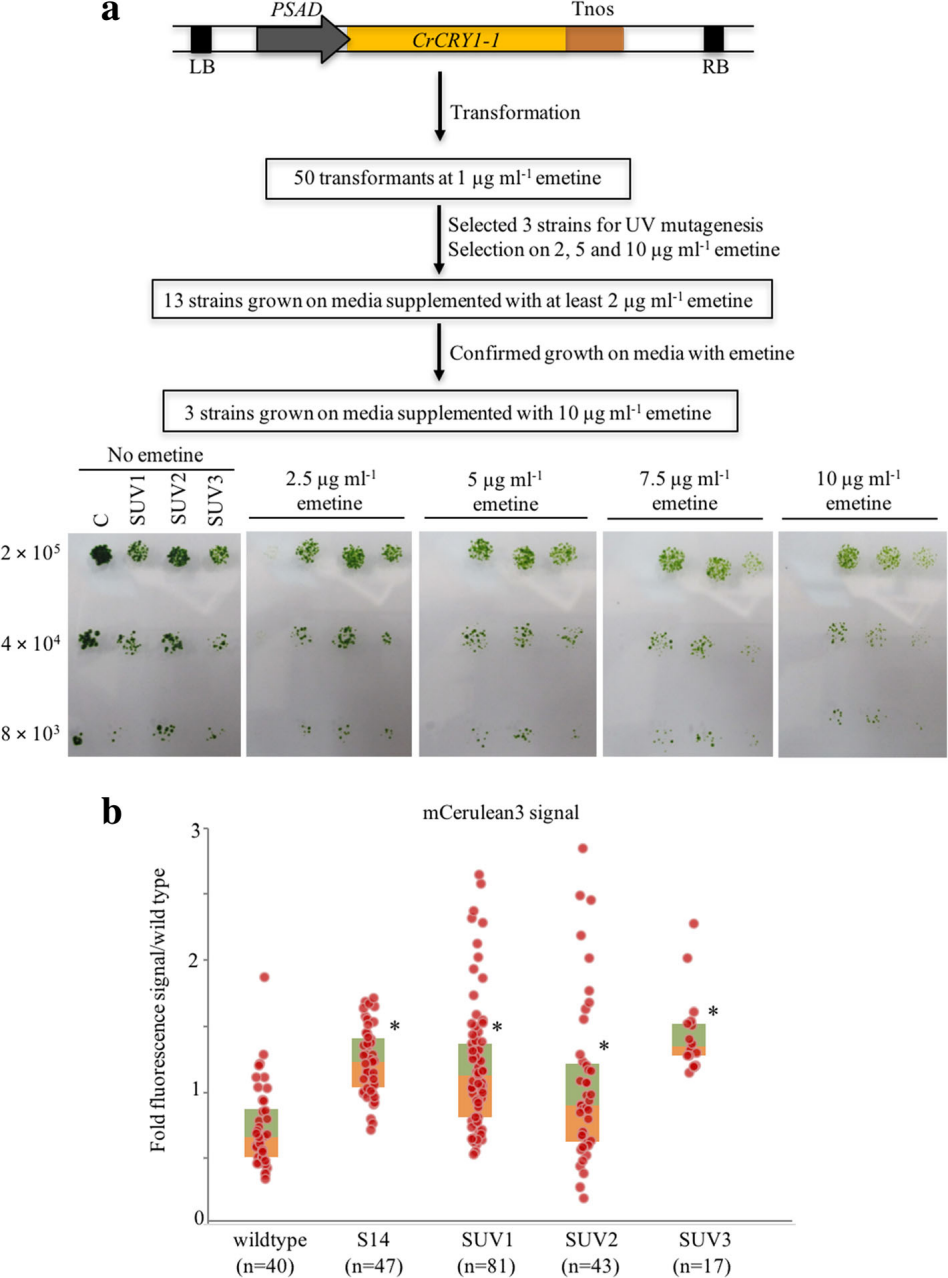
UV mutagenesis and selection of SUV strains. **a** *CrCRY1-1* expression construct and the experimental procedure for selecting transformants. The lower panel shows three selected strains, SUV1-3, that grew on the medium supplemented with emetine up to 10 μg ml^− 1^ compared to the TISTR8447 wild type. **b** Box plots overlaid with scatter plots of mCerulean3 fluorescence signals of transformants generated from wild type, S14 (a *CrCRY1-1* expressing strain) and SUV1-3. (n) indicates the number of transformants for each strain. Asterisks indicate significant differences between signals from transformants generated from wild type and others by Kruskal-Wallis Test (*p* < 0.05)

To further improve the transgene expression, TISTR8447 mutants with enhanced transgene expression were generated using UV mutagenesis and a selection strategy using *CRY1-1* (conferring emetine resistant) as reported by Neupert et al. [21]. Transformation of the TISTR8447 using pCRE-*CRY1-1* was selected on the medium supplemented with 1 μg ml^− 1^ emetine. Fifty selected transformants were tested on media supplemented with 1–20 μg ml^− 1^ emetine to confirm that they could grow on the medium supplemented with at most 1 μg ml^− 1^ emetine. Three transformants (S14) were chosen for UV mutagenesis at 0.1% survival rate and subsequently selected using 2, 5 and 10 μg ml^− 1^ emetine. Finally, three mutants (SUV1, 2 and 3), which were able to grow on 10 μg ml^− 1^ emetine, were obtained (Fig. 3a). These mutants were then tested for any improvement on transgene expression by transformation using pOPT-*mCerulean3* followed by fluorescence microplate analysis (Fig. 3b). Although the highest levels of the fluorescence signal ratios were in the range of 2- to 3-fold, which were similar to transformants of control strains (S14), the SUV1-3 provided overall higher frequencies of transformants with high fluorescence signal ratios (8/81 or 10%, 5/43 or 12% and 2/17 or 12% for SUV1-3, respectively). This result demonstrates that although the level of transgene expression is only moderately improved, these TISTR8447 mutants provide increases in the frequency of transgene expressed transformants.

### Improving transgene expression by generating TISTR8447 white mutants and *PSY::E2A* transcript fusion

**Fig. 4:**
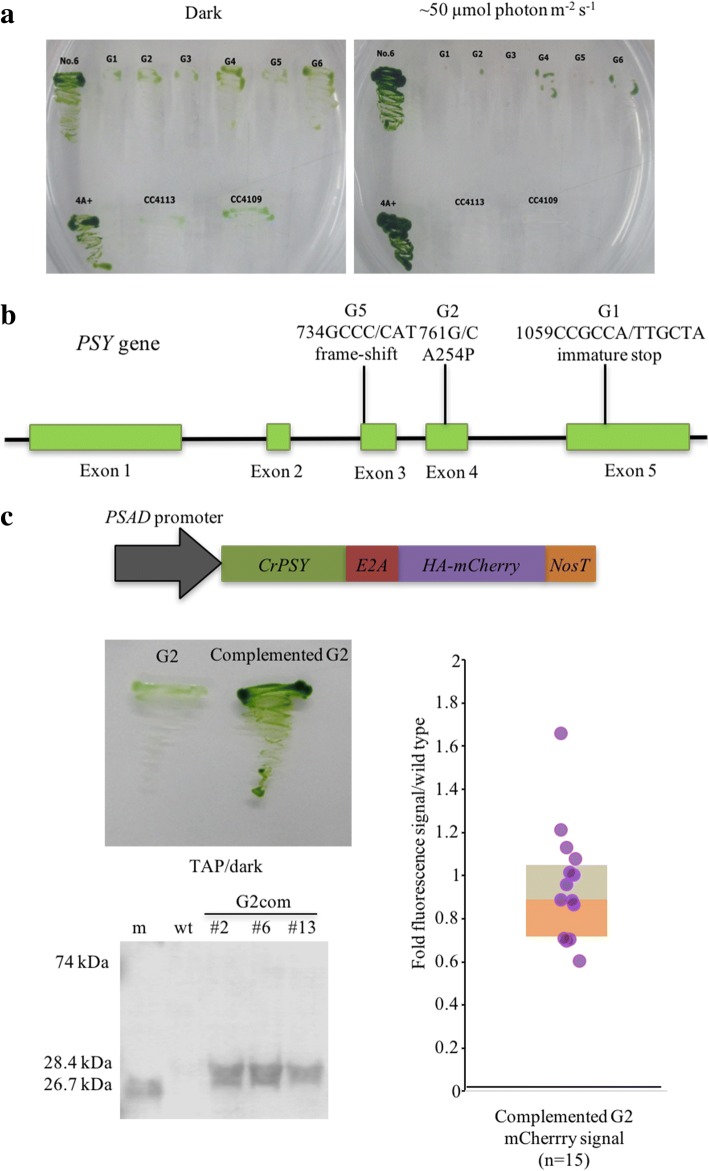
Generation of *psy* white mutants and complementation using *CrPSY*. **a** Six isolated white mutants (G1-6) on TAP medium under light and dark conditions (No.6: TISTR8447, 4A+: *Chlamydomonas*, cc4113 and cc4109: *Chlamydomonas psy* white mutants). **b** Genotyping of *psy* mutants by direct PCR and sequencing. **c** Complementation of *psy* white mutants using *CrPSY* coding sequence fused in-frame with *E2A* and *mCherry.* mCherry was detected by both fluorescence microplate reader and immunoblot analysis (m: mCherry from *E. coli*, wt: TISTR8447). mCherry carrying HA tag in the algae is 28.4 kDa, while that in *E. coli* is 26.7 kDa. A fainted band representing 74 kDa of CrPSY::E2A::mCherry fusion protein was observed (see Additional file 8 for longer exposures and Additional file 10 for the full length blot)

The transcript fusion system using *ble::E2A* have been shown so far to be the best improvement of transgene expression in TISTR8447, but the use of zeocin as a selective pressure is impractical for large-scale culture and affects the algal growth. To further improve this, an alternative expression strategy was developed by using *Phytoene synthase* (*PSY*) mutants, *PSY* gene complementation and light as a selective pressure. *Chlamydomonas* with *PSY* mutations were previously shown to exhibit pale-green or whitish colony, perish under the light as low as 8 μmol photons m^− 2^ s^− 1^ and to be complemented by transformation using *PSY* gene [38]. By adopting this idea, white mutants of the TISTR8447 were generated by UV mutagenesis and selection of pale-green colonies grown in the dark. From 50 selection plates, each with 10^6^ cells, six white mutants, named G1-G6, were obtained after three rounds of subcultures in the dark (Fig. 4a). Four white mutants (G1, G2, G3 and G5) ceased growth under light at 50 μmol photons m^− 2^ s^− 1^, similar to the cc4113 and cc4109 *Chlamydomonas psy* white mutants [38], while G4 and G6 mutants were able to grow under the light. After sequencing the *PSY* gene in these mutants, point mutations were observed in the *PSY* coding sequence in G1, G2 and G5 mutants (Fig. 4b), but not in G3. The G1, G2 and G5 *psy* mutants were then tested for complementation using a construct containing *Chlamydomonas PSY* coding sequence fused with *E2A* and *HA-mCherry*. Transformation of these three white mutants using the *Agrobacterium* method was unsuccessful, and this may be because the mutant grew very slow and could not out-compete *Agrobacterium* growth. Electro-transformation yielded complementation from G1 and G2 mutants with green colonies after selection under light at 50 μmol photons m^− 2^ s^− 1^ for 7–10 days (Fig. 4c). However, the transformation rates were extremely low, approximately 1–4 CFU per 5 × 10^6^ cells per plate.

The G2 complemented strains were analyzed by fluorescence microplate reader and showed that most of the transformants had low levels of the fluorescence signals for mCherry (Fig. 4c). The frequency of the transgene expressers (up to a 2-fold signal ratio) was one among 15 transformants (~ 7%), somewhat similar to that of intron-containing vectors. Unlike that of *ble::E2A* vectors with the zeocin selection, the expression of *PSY::E2A* was still low. This may be because the PSY functions as an enzyme in carotenoid biosynthesis pathways, while ble protein functions by binding to zeocin in a direct proportion [23]. Therefore, light selection could not drive the *PSY* expression to the levels achieved by zeocin selection. Spontaneous revertants were also monitored in the transformation controls (without plasmid) and, so far, there were only two revertants observed among 40 selection plates (5 × 10^6^ cells per plate). To preclude the possibility of these complemented strains being derived from revertants, mCherry expression was confirmed by immunoblot analysis (Fig. 4c). The non-cleaved PSY::E2A::HA-mCherry fusion protein was observed at a very low level indicating that the E2A peptide was efficiently self-cleaved in the TISTR8447 (Additional file 8). This result demonstrates that *psy* white mutants of TISTR8447 can be used as host cells for transformation using *PSY* selectable marker and light selection. However, the expression levels of the *PSY::E2A* transgene were in the same range as that of constructs containing introns and far lesser than those using SUV mutants and *ble::E2A* with the zeocin selection, respectively.

### Growth and total lipid contents of SUV mutants and complemented white mutants

**Fig. 5:**
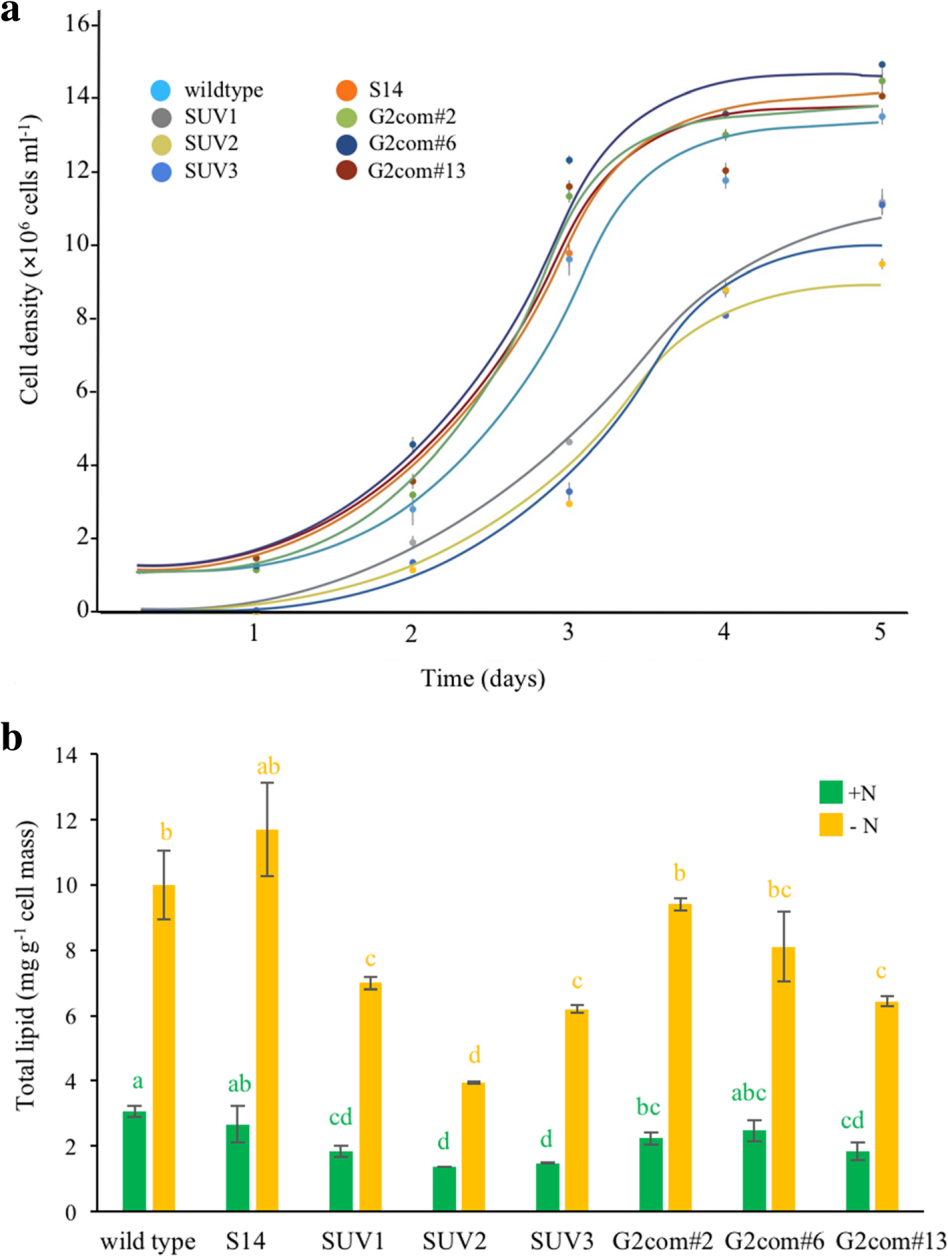
**a** Growth curves of S14, SUV1-3 and complemented G2 strains compared to TISTR8447 wild type. **b** Total lipid content of the microalgae in N-supplemented and N-deprived media grown for 3 days. Error bars represent standard deviation (*n* = 6), and bars with the same letter are not significantly different (Tukey’s test, *p* < 0.05)

To examine whether mutations for SUV and white mutants had any effect on growth or lipid production, the mutants were analyzed for growth curves and total lipid contents. While the growth of three complemented G2 strains and S14 was similar to that of the wild type, SUV1-3 grew slower (Fig. 5a). The cell growth of SUV1-3 was limited to approximately 10 **×** 10^6^ cells ml^− 1^, whereas the wild type could grow up to 15 **×** 10^6^ cells ml^− 1^. The lipid contents of S14 and G2 complemented strains in both N-supplemented and N-deprived media were similar to those of wild type, but those of SUV1-3 were significantly lower for both media (*p* < 0.05) (Fig. 5b). This result indicates that mutations, which provide improved transgene expression in SUV mutants, also affect physiological processes of the TISTR8447 resulting in slow growth and less accumulated lipids.

## Discussion

This work shows that *Agrobacterium-*mediated transformation is a convenient method for simultaneously testing the transformation of many microalgae. In this work, ten strains were tested, and six of them were successfully transformed. The best transformation rate was obtained from *S. acutus* TISTR8447 at 217.5 ± 75 CFU per 10^6^ cells, and this was selected as our best candidate for evaluating the transgene expression. *Coelastrum, Chlorococcum, Monoraphidium* and *Tetradesmus* are reported for their first trial here for the *Agrobacterium* method. Noting that although *M. braunii*, *A. falcatus, A. densus* and *Coelastrum* spp. were unable to be transformed in this work, this should not preclude their possibility to be transformed by other means. *Agrobacterium-*mediated transformation of microalgae was first introduced by Kumar et al. [30] for a successful transformation in *Chlamydomonas*, and various protocols have been developed since then to improve its efficiency [9, 39, 40]. It was then applied to many other microalgal species including *Haematococcus pluvialis* with the transformation rate at 153 ± 4 CFU per 10^6^ cells [41], *Dunaliella bardawil* at 39–42 CFU per 10^6^ cells [42], *Schizochytrium* at 50–170 CFU per 10^6^ cells [43], *Scenedesmus almeriensis* at 90 CFU per 10^6^ cells [26], *Dunaliella salina* at 40 ± 5 CFU per 10^6^ cells [29] and *Tetraselmis chuii* at 150 ± 90 CFU per 10^6^ cells [27]. And, with a different unit of transformation rate, *Nannochloropsis* sp. [44], *Chlorella vulgaris* [45] and *Ankistrodesmus* sp. [46] were shown to be transformed based on *GUS* expression at approximately 21, 30 and 3.5% efficiency, respectively. Taken together, this work provides support for the use of the *Agrobacterium*-mediated method for transforming other potential industrial microalgae yet to be tested.

Extremely inefficiently transgene expression is commonly observed in microalgae [9], and this appears to be the case for *Scenedesmus* as indicated by this and previous works. For example, transformation of *S. almeriensis* yielded little or very low GUS reporter expression even after screening many transformants [26], that of *S. bajacalifornicus* had only 2.96% of transformants with detectable GUS staining [46] and that of *S. obliquus* also showed only 1.5% of the transformants with *GFP* expression [25]. In our case, no *GFP* expression was observed at neither transcript nor protein level. Our attempt to improve the transgene expression in TISTR8447 by using tools based on *Chlamydomonas*, so far, shows that the transcript fusion using *ble::E2A* provided the highest improvement with up to 10-fold increases of mCherry expression and up to 85% of transformants with observable mCherry. However, other strategies including the uses of intron-containing constructs, SUV1-3 mutants and white mutants with *PSY::E2A* complementation provided only minor improvements with at most 2–4-fold increases of the reporter expression. The majority of the transformants from the intron-containing constructs and the white mutants had very low transgene expression, while SUV mutants had increases in the frequency of transgene expressors.

Transcriptional gene silencing was proposed to be the key factor that causes the low transgene expression in *Chlamydomonas* [13, 19, 21, 36, 47] and most likely in many other microalgae [48–50]. Noting that a recent work by Mini et al. [9] reported that rearrangements of transgenes could also be another factor contributing to the inefficient expression in *Chlamydomonas*. Because gene expression tools that could circumvent the gene silencing effects were successfully developed in *Chlamydomonas*, these tools could be applied to other microalgae, particularly in the TISTR8447. However, the improvements were far less than those obtained in *Chlamydomonas.* The development of the *ble::2A* systems provided more than a 100-fold improvement of transgene expression in *Chlamydomonas* [23, 34, 35, 37], while it was only a 10-fold increase in the TISTR8447. Insertions of an endogenous intron(s) into transgenes or the surrounding 5′ and 3′ untranslated regions led to improved transgene expression in *Chlamydomonas* [51–53]. In contrast, these *Chlamydomonas* introns only provided small increases in the transgene expression in the TISTR8447. A little improvement in the TISTR8447 observed here by the use of the introns could be because of the difference in intron splicing between the species, or these introns could only occasionally help the transgene escaping the silencing controls in the TISTR8447. Perhaps, *Scenedesmus* might have a more robust gene silencing mechanism than that in *Chlamydomonas*.

Neupert et al. [21] first reported the strategic isolation of *Chlamydomonas* mutants with improved transgene expression using *CRY1-1* and elevated emetine concentrations for selection. The selected mutants, UVM4 and UVM11, had demonstrated up to 100-fold increases in emetine resistant. Less than expected, our selections of mutagenized TISTR8447 yielded up to a 10-fold increase in emetine resistance, and the expression levels based on the fluorescence ratios were improved only up to a 2-fold. Nonetheless, the SUV1-3 had higher frequencies of transgene expressors, in which transformants with at least a 2-fold signal could be accounted for ~ 10–12% compared to ~ 2.5% observed in wild type. This improvement is somewhat comparable to UVM4 and UVM11 that provided 4.5-fold increases in the frequency of transgene expressors compared to the wild type [47].

MET1 (DNA methyltransferase1) is one of the key enzymes for the DNA methylation transgene silencing along with other DNA-methylation independent gene silencing enzymes [13]. *Chlamydomonas met1* insertional mutants were shown to provide an improvement for transgene expression. Work from Kurniasih et al. [13], which performed UV mutagenesis using the *met1* mutant and selection using Neupert et al. [21] selection method, yielded mutants with even higher transgene expression. Because only moderate improvement was achieved in the SUV mutants, it is likely that there are still other transcriptional silencing genes present in the TISTR8447. Further mutations in the transcriptional silencing related genes may be required for further improvement.

Mutations that provide improved transgene expression in *Chlamydomonas* are known to be associated with epigenetic controls, and these may affect cell growth, as demonstrated in *Chlamydomonas* UVM mutants of the *met-1* insertional mutant [13]. Indeed, mutations of SUV1-3 are most likely related to epigenetic control machinery, and they affect both cell growth and lipid production, as none of these effects was observed in the S14 control strain. This result shows that while the SUV1-3 mutants have gained some improvement on the transgene expression, their growth and ability to accumulate lipids were weakened, compromising cell mass and lipid production. This result confirms the previous observation of the negative aspect of using mutants related to epigenetic controls. Thus, cautions should be made before performing genetic screening for enhancing transgene expression, especially when the cell performances are the key to the application. Nonetheless, the white mutants, when complemented, could provide sufficiently intact growth and have only slight decreases in the lipid content. The result also suggests that while *CrPSY* can sufficiently complement the *SaPSY* knockout, yielding complemented strains with robust cell growth, it could not provide the lipid accumulation as efficient as the *SaPSY*.

Even though the *PSY::E2A* construct is not as effective as *ble::E2A* in forcing expression, this system provides an alternative selectable marker for microalgae. Current selectable markers used for microalgae transformation are based on antibiotic selection, for example, bleomycin/zeocin resistance gene [33] and paromomycin resistance gene [54]. Recently, the herbicide norflurazon was recently developed for transformation selection using *Phytoene desaturase* (*PDS*) with an amino substitution L505F in some microalgae [55–57]. The use of *PSY* gene as a selectable marker for microalgae, so far, has not been reported. This may be because of two main reasons: white mutants needed to be generated by mutagenesis and screened for each microalga, and white mutants are weak with slow growth and difficult to transform. For the work presented here, this is one of the most economical transgene selection approaches as only a light source is required. This selectable marker is compatible with an outdoor and large-scale culture as transgene constructs can be maintained by sunlight.

To this end, among the *Chlamydomonas* gene expression tools tested, the transcript fusion using *ble::E2A* appeared to be the most efficient and could instantaneously mitigate the inefficient transgene expression in microalgae, in particular, the TISTR8447. However, the exposure of zeocin antibiotic to the algal cells generally results in cell toxicity and affects cell growth and potentially the production of products and yields [58]. Furthermore, the use of zeocin is uneconomical for large-scale production, limiting the use of *ble::E2A* system. A consensus for inefficient transgene expression has been evidenced in many oleaginous microalgae and *Scenedesmus* species as reported here. The challenges in gaining high transgene expression in microalgae are the critical bottleneck for current development in Eukaryotic microalgal biotechnology. Further improvements and newly developed tools are critically needed to allow efficient use of microalgae for biotechnology application.

## Conclusions

This work demonstrates that oleaginous microalgae isolated from natural resources can be screened for selecting strains with high transformation efficiency, but they generally have a low capacity for transgene expression. A selected strain, *S. acutus* TISTR8447, was tested to improve the transgene expression using strategies developed in *Chlamydomonas*, but overall improvement was of moderate for both levels and frequency of expression. So far, the most effective tool giving the highest improvement is the transcript fusion using *ble*::*E2A* system. Furthermore, a new strategy for transgene expression has been developed using *psy* white mutants with an expression vector containing *PSY*::*E2A* for complementation and light selection. Finally, this work demonstrates that genetic engineering of non-model microalgae is still a challenging task. New tools and strategies are critically needed for transgene expression in promising industrial microalgae.

## Methods

### Microalgae and cultivation conditions

Microalgae were obtained from Thailand Institute of Science and Technological Research (TISTR) including *Coelastrum* sp. (TISTR8511), *Scenedesmus acutus* (TISTR8555), *S. acutus* (TISTR8540), *S. acuminatus* (TISTR8519), *S. acutus* (TISTR8447), *Chlorococcum humicola* (TISTR8434), *Monoraphidium braunii* (TISTR8429), *Ankistrodesmus falcatus* (TISTR8557), *Tetradesmus cumbricus* (TISTR8480), *Ankistrodesmus densus* (TISTR8505). These accessions were chosen based on their potential high accumulation of lipid droplets under the N deprivation condition and the variation of algal species. *Chlamydomonas* white mutants CC4113 (Its1-207 mt+) and CC4109 (Its1-203 mt+) were obtained from Chlamydomonas Resource Center. Microalgae were cultured in either Tris-acetate phosphate (TAP) or BG-11 media at 25 °C under continuous light (50 μmol photons m^− 2^ s^− 1^), excepting that those white mutants were incubated in the dark. The N-deprived medium (TAP-N) was prepared by substituting KNO_3_ with KCl. For growth analysis, three cultures of 50 ml were started at the of 10^6^ cells ml^− 1^ cell density, and 1 ml sample was collected daily to monitor for cell density.

### Plasmid constructions

pOPT vector series with reporter genes including *mRuby2*, *mCerulean3*, *mVenus* and *Clover* [22] were obtained from Chlamydomonas Resource Center. pCXSN-GFP was constructed by TA cloning using the *Agrobacterium* transformation vector pCXSN [59] and *GFP* coding sequence, which was PCR amplified using GFP-F and GFP-R primers (Additional file 9). Transcript fusion vectors were constructed using pPLV02 [60] as the vector backbone. The pPLV02 backbone was amplified using pplv-F and pplv-R primers. PSAD promoter, RBSC2 promoter-*ble* with introns and *ble* without intron fragments were amplified from pSL18 [61, 62] using psad-F and psad-R primers, pSP124S [51] using rbcs-F and Zeo-E2A-R primers and pICZ (Thermo Fisher) using Zeo-F and Zeo-E2A-R primers, respectively. Each primer has an adaptor sequence for subsequent sequence and ligation independent cloning (SLIC) [63]. All PCR cloning was performed using hi-fidelity Q5 DNA polymerase (New England Biolabs, USA). pCreZ-*E2A* and pCreZint-*E2A* were constructed by fusing the pPLV02 PCR fragment with PSAD promoter and *ble* without intron, and RBSC2 promoter-*ble* with introns, respectively, by SLIC technique. *HA*-*mCherry* was subsequently inserted into both vectors at NcoI site by PCR amplification using HA-mcherry-F and mcherry-R primers and SLIC.

For *CrCRY1-1* expression construct, the coding region of *CrCRY1* was PCR amplified from *Chlamydomonas* cDNA using gene specific primers, before being re-amplified using a reverse primer with a modified base at the last codon from CTG to CCG (cry1-1-F and cry1-1-TtoC-R primers) to generate *CrCRY1-1* coding sequence. The *CrCRY1-1* fragment with adaptor sequences was then inserted into pCreZ-*E2A* at EcoRI and BamHI sites by SLIC, removing *ble*-*E2A*. For *CrPSY::E2A::mCherry* constructs, the *CrPSY* coding sequence was PCR amplified from *Chlamydomonas* cDNA and re-amplified using crpsy-F and crpsy-R primers, which contain adaptor sequences for SLIC. The *CrPSY* fragment was then ligated to a vector fragment, which was amplified from pCreZ-*E2A* by E2A-F and psad-crpsy-R primers, replacing *ble* coding sequence and in frame with *E2A*. Plasmids for *Agrobacterium-*mediated transformation were transferred into *Agrobacterium* cells by electroporation.

### Transformation

The microalgal strains were tested for their sensitivity to hygromycin B before performing an *Agrobacterium*-mediated transformation. Briefly, 10^6^ cells were spread onto TAP or BG-11 agar media supplemented with 10–100 μg ml^− 1^ of hygromycin B for 14 days. This sensitivity test was performed in triplicate. For *Agro*-transformation, microalgae were cultured in liquid media for 3–4 days, and 10^6^ cells were grown on solid media for 2–3 days until forming a lawn. *A. tumefaciens* EHA105 harboring transformation plasmids were grown in LB media supplemented with 25 μg ml^− 1^ of rifampicin and 50 μg ml^− 1^ of kanamycin under continuous shaking at 28 °C for 2 days. For evaluation of *Agrobacterium* strains, A41, GV3101 and LBA4404 strains were used in replacement of EHA105. The algal lawn was mixed with 100 μl of *Agrobacterium* (OD_600_ = 1) using a spreader and co-cultivated in the dark for 2 days before transferring onto solid TAP or BG-11 selective media supplemented with 250 μg ml^− 1^ cefotaxime and hygromycin B at specific concentrations. Colonies obtained from selection plates were subcultured twice on the selection media before being designated as transformants. Transformation rates were calculated based on the numbers of resistant colonies per 10^6^ starting cells. *Agrobacterium* cells without expression plasmid were used for transformation as negative controls.

For electro-transformation, log phase cells at no more than 2 × 10^6^ cells ml^− 1^ were harvested by brief centrifugation at 400×g and resuspended in TAP supplemented with 40 mM sucrose (TAP sucrose) at 2 × 10^8^ cells ml^− 1^. Thirty-eight microlitres of the algal suspension were mixed with 2 μl of a plasmid vector (400 ng) before transferring to a 2 mm gap electroporation cuvette and pulsing using Bio-Rad Gene Pulser II with the following condition: 0.4 kV, 25 μF and 500 Ω. The algal cells were immediately transferred to 10 ml TAP sucrose and cultured at the normal condition with constant shaking at 100 rpm under low light for an overnight before spreading onto TAP agar supplemented with 30 μg ml^− 1^ of paromomycin or 10 μg ml^− 1^ of zeocin for 7–10 days.

For the transformation of white mutants, a white colony was cultured in TAP sucrose with constant shaking in the dark up to 10 days or until reaching 1–2 × 10^6^ cells ml^− 1^. Cells were harvested and resuspended in 40 mM sucrose TAP at 2 × 10^8^ cells ml^− 1^. Subsequently, 38 μl of the suspension was mixed with 2 μl of a plasmid before being electroporated using the same condition as above and cultured in 10 ml TAP sucrose for overnight with constant shaking in the dark. The cells were plated onto TAP agar and incubated in the dark for 5 days and then under light (50 μmol photons m^− 2^ s^− 1^) for 7–10 days.

### UV mutagenesis and mutant screening

UV mutagenesis was performed by irradiating the TISTR8447 at a density of 5 × 10^6^ cells ml^− 1^, which were previously kept in the dark for 2 h, in a petri dish using a UV transilluminator for 2 h to obtain a 0.1% survival rate. Cells were cultured overnight with shaking in the dark before plating onto TAP agar medium at 10^6^ cells per plate. For SUV mutants, *CRY1-1* transformants were UV irradiated and plated onto TAP agar medium supplemented with emetine at 2, 5 and 10 μg ml^− 1^. For white mutants, irradiated cells were plated onto TAP agar medium and incubated in the dark for 14 days. Colonies with pale-green were subcultured in the dark.

### Molecular characterization

For DNA and RNA isolation, the algal cells were subjected to a mechanical disruption in liquid nitrogen using a mortar and pestle. DNA was isolated using the CTAB method, whereas total RNA was isolated using GENEzol TriRNA Pure Kit (Geneaid, Taiwan). RNA was purified by using RNase-free DNase and RNA Cleanup Kit. cDNA was synthesized using one microgram of total RNA by MMuLV reverse transcriptase. PCR and RT-PCR reactions were performed using *Taq* DNA polymerase and primers listed in Additional file 9. For sequencing of the *PSY* coding sequence of TISTR8447 (Genbank accession MF401544), *SaPSY* of the white mutants was amplified from total DNA using hi-fidelity Q5 DNA polymerase and sapsy-seq-F and sapsy-seq-R primers and sequenced.

For western analysis, a total protein was isolated by grinding the cells with liquid nitrogen and resuspended in a protein extraction buffer [20 mM Tris-HCl pH 8.0, 150 mM NaCl, 2 mM EDTA, 10% (*v*/v) glycerol, 0.5% IGEPAL CA-360 and 1 mM PMSF]. The total protein was quantified by Bradford assay before subjecting to SDS-PAGE and blotting onto a PVDF membrane. mCherry was detected using rabbit anti-mCherry as a primary antibody at 1:1000 dilution and anti-rabbit-HRP as a second antibody at 1:10,000 dilution. The western signal was detected using WesternSure Premium Chemiluminescence Substrate (Li-COR, USA) and C-DiGit Blot Scanner (Li-COR, USA).

### Fluorescence detection

Fluorescent reporter signals were analyzed using a microplate reader. The excitation and emission wavelengths are as follows; mRuby2 (ex = 558 nm, em = 605 nm), mCerulean3 (ex = 445 nm, em = 503 nm), mVenus (ex = 515 nm, em = 550 nm), Clover (ex = 477 nm, em = 515 nm), mCherry (ex = 562 nm, em = 607 nm). Chlorophyll autofluorescence was measured at 440 nm for excitation and 685 nm for emission. Standard curves for the conversion of fluorescence signal to protein contents were generated from recombinant GST-mCerulean and GST-mCherry purified from *E. coli* and quantified using Bradford assay. The protein concentrations used was 1.25–400 ng μl^− 1^ in the TISTR8447 algal culture at 10^6^ cells ml^− 1^. The chlorophyll fluorescence was used for calculating the cell number based on a standard curve between the chlorophyll signals and cell numbers. Selected transformants with a high level of expression of transgenes were then assessed for fluorescence signal using Nikon C2 Si confocal microscopy.

### Stability test

Transformants were maintained on media without hygromycin B for 2 weeks before subculturing onto media with and without hygromycin B to determine the retention of the selectable marker gene without the selection pressure. For the G2 complemented lines, the dark and light conditions were used instead of the antibiotic. These subcultures were repeated every 2 weeks.

### Lipid quantification using vanillin method

This lipid quantification using vanillin was modified from Mishra et al. [64]. Cells were grown in a starting culture using liquid TAP medium for 2–3 days (~ 2 × 10^6^ cells ml^− 1^ concentration) before dividing in half, and the medium of each half was replaced by liquid TAP or TAP-N at 2 × 10^6^ cells ml^− 1^ concentration before culturing for 3 days. Approximately 1.5 × 10^6^ cells with known mass was mixed with 200 μl sulfuric acid, incubated at 95 °C for 10 min before placing on ice for 5 min, added with 500 μl phospho-vanillin [0.12% (*w*/*v*) vanillin in 80% (*v*/v) phosphoric acid] and incubated at 37 °C for 15 min. The solution was briefly centrifuged at 3000 rpm for 5 min, and the absorbance at 530 nm of the sample was assessed. Total lipid was quantified against the standard curve generated from canola oil.

### Nile red staining

Microalgae were stained using Nile red (10 μg ml^− 1^ in DMSO) and Calcofluor (1%) for 30 min in the dark at 25 °C. The samples were then fixed in 4% paraformaldehyde before washing twice with PBS solution and analyzed using Nikon C2 Si confocal microscopy (543 nm excitation, 525–605 nm emission).

### Statistical analysis

The statistical tests used for analyzing the data varied based on the distribution of each data set. Kruskal-Wallis test was conducted to assess the difference in the transformation efficiency of different *Agrobacterium* strains and the levels of fluorescence signals among populations of microalgal strains. For datasets with a normal distribution, one-way analysis of variance (ANOVA) using an alpha of 0.05 and a multiple range test using Tukey’s method was conducted.

## Additional files

Additional file 1: Survival of ten microalgal strains on different concentrations of hygromycin B. (PDF 37 kb)

Additional file 2: Representative cultured and selective plates for *Agrobacterium*-mediated transformation of TISTR8540. (PDF 552 kb)

Additional file 3: A transgene stability test of TISTR8540 and TISTR8447. (PDF 1536 kb)

Additional file 4: Nile red staining of the TISTR8447 after 3 days of nitrogen starvation. (PDF 101 kb)

Additional file 5: Transformation rates of the TISTR8447 using four *Agrobacterium* strains. (PDF 39 kb)

Additional file 6: *Agrobacterium*-mediated transformation of *S. acutus* TISTR8447. (PDF 446 kb)

Additional file 7: The schematic of pCreZ vectors. (PDF 64 kb)

Additional file 8: Western analysis of CrPSY::E2A::mCherry fusion protein with extended exposure times. (PDF 127 kb)

Additional file 9: Oligonucleotide primer sequences. (PDF 111 kb)

Additional file 10: The full length immuno blot of G2 complemented strains. (PDF 170 kb)

## Abbreviations

ANOVA: Analysis of variance
aphIV: Hygromycin phosphotransferase
BG11: Blue-green Medium
ble: Bleomycin resistant protein
CaMV: Cauliflower mosaic virus
CFU: Colony forming unit
CrPSY: *Chlamydomonas reinhardtii* phytoene synthase
CRY1-1: Cryptochrome circadian clock 1-1
CTAB: Cetyltrimethylammonium bromide
DMSO: Dimethyl sulfoxide
EDTA: Ethylenediaminetetraacetic acid
GFP: Green fluorescence protein
GST: Glutathione S-transferase
GUS: Beta-glucuronidase
HA: Hemagglutinin
HRP: Horseradish peroxidase
MET1: DNA methyltransferase1
MMuLV: Reverse transcriptase: moloney murine leukemia virus reverse transcriptase
PDS: Phytoene desaturase
PMSF: Phenylmethylsulfonyl fluoride
PSY: Phytoene synthase
PVDF: Polyvinylidene fluoride
SaPSY: *Scenedesmus acutus* phytoene synthase
SDS-PAGE: Sodium dodecyl sulfate polyacrylamide gel electrophoresis
TAP: Tris-acetate phosphate buffer
TAP-N: Tris-acetate phosphate buffer without nitrogen
Taq polymerase: *Thermus aquaticus* polymerase
UV: Ultraviolet
