# Supplementary material for: Evaluation of strategies for improving the transgene expression in an oleaginous microalga Scenedesmus acutus
Source: BMC Biotechnol. 2019 Jan 10;19:4. doi: 10.1186/s12896-018-0497-z (PMC6327543; doi:10.1186/s12896-018-0497-z)
Supplement: Supplementary file 1 — Survival of ten microalgal strains on different concentrations of hygromycin B. (PDF 37 kb) [file 12896_2018_497_MOESM1_ESM.pdf]

Additional file 1

Additional file 1**.** Survival of ten microalgal strains on different concentrations of hygromycin B. (+++) indicates more than 1,000 CFU per plate and (++) indicates 10-100 CFU per plate.

Species	Hygromycin B (μg ml^{-1})					
	0	10	20	30	40	50
Coelastrum sp. (TISTR 8511)	+++	-	-	-	-	-
S. acutus (TISTR 8555)	+++	+++	-	-	-	-
S. acutus (TISTR 8540)	+++	+++	+++	-	-	-
S. acuminatus (TISTR 8519)	+++	+++	+++	-	-	-
S. acutus (TISTR 8447)	+++	+++	-	-	-	-
C. humicola (TISTR 8434)	+++	+++	-	-	-	-
M. braunii (TISTR 8429)	+++	-	-	-	-	-
A. falcatus (TISTR 8557)	+++	-	-	-	-	-
T. cumbricus (TISTR 8480)	+++	+++	++	++	-	-
A. densus (TISTR 8505)	+++	-	-	-	-	-
