# Supplementary material for: Evaluation of strategies for improving the transgene expression in an oleaginous microalga Scenedesmus acutus
Source: BMC Biotechnol. 2019 Jan 10;19:4. doi: 10.1186/s12896-018-0497-z (PMC6327543; doi:10.1186/s12896-018-0497-z)
Supplement: Supplementary file 2 — Representative cultured and selective plates for Agrobacterium-mediated transformation of TISTR8540. (PDF 552 kb) [file 12896_2018_497_MOESM2_ESM.pdf]

Additional file 2

S. acutus TISTR 8540

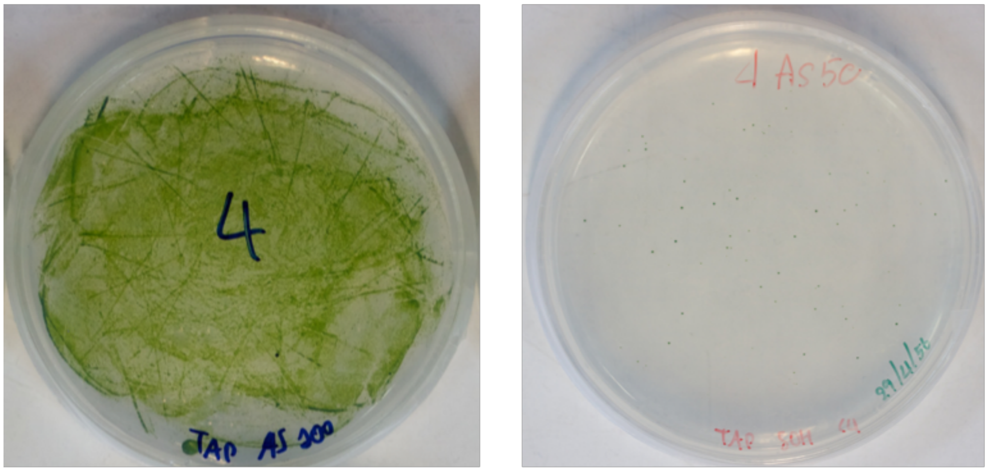

A lawn culture of 7 day-old on TAP supplemented with 100 µM Acetosyringone

Transformation selection on TAP supplemented with 250 µg ml^{-1} cefotaxime and 50 µg ml^{-1} hygromycin B for 10 days

Additional file 2. Representative cultured and selective plates for *Agrobacterium*-mediated transformation of TISTR8540. The left panel shows a lawn culture before co-cultivation with *Agrobacterium,* and the right panel shows transformant colonies on a selective plate.
