# Supplementary material for: Evaluation of strategies for improving the transgene expression in an oleaginous microalga Scenedesmus acutus
Source: BMC Biotechnol. 2019 Jan 10;19:4. doi: 10.1186/s12896-018-0497-z (PMC6327543; doi:10.1186/s12896-018-0497-z)
Supplement: Supplementary file 3 — A transgene stability test of TISTR8540 and TISTR8447. (PDF 1536 kb) [file 12896_2018_497_MOESM3_ESM.pdf]

Additional file 3

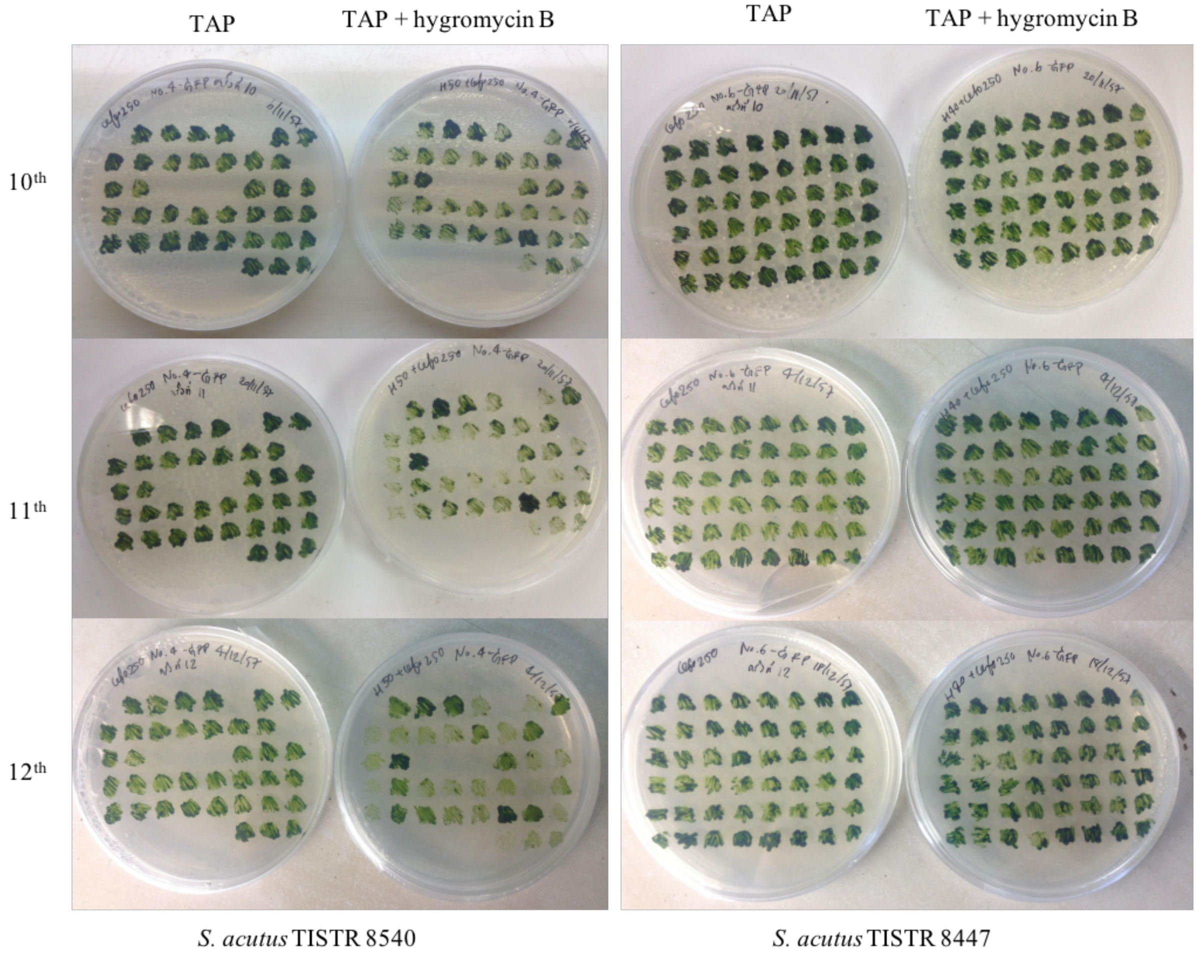

Additional file 3. A transgene stability test of TISTR8540 and TISTR8447. Transformants from each strain were continuously cultured on TAP medium with weekly subculturing and testing on TAP medium supplemented with hygromycin B up to 12 rounds.
