# Supplementary material for: Evaluation of strategies for improving the transgene expression in an oleaginous microalga Scenedesmus acutus
Source: BMC Biotechnol. 2019 Jan 10;19:4. doi: 10.1186/s12896-018-0497-z (PMC6327543; doi:10.1186/s12896-018-0497-z)
Supplement: Supplementary file 4 — Nile red staining of the TISTR8447 after 3 days of nitrogen starvation. (PDF 101 kb) [file 12896_2018_497_MOESM4_ESM.pdf]

Additional file 4

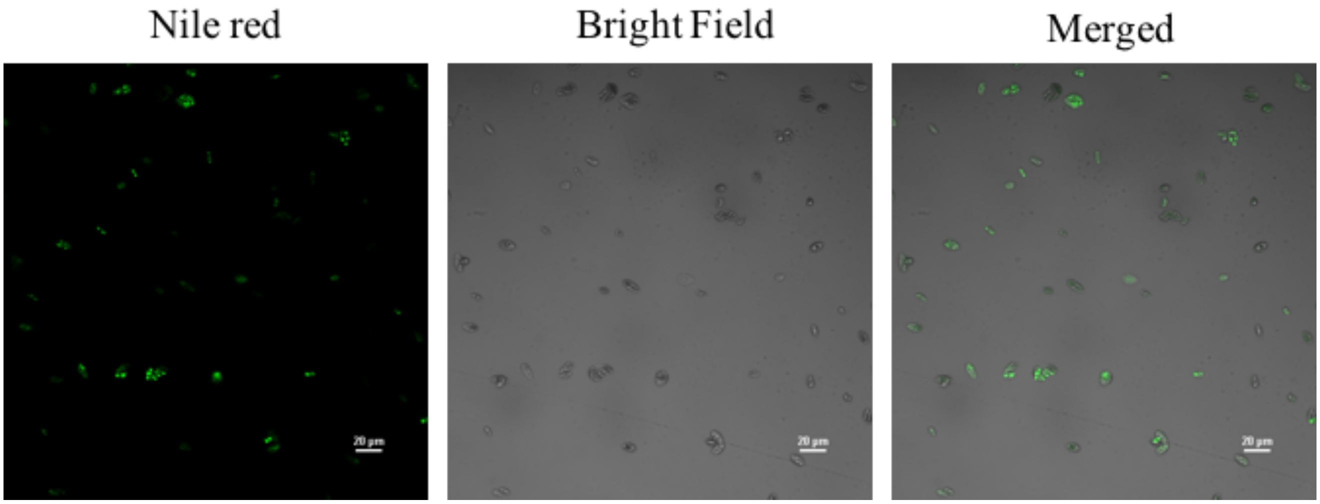

Additional file 4. Nile red staining of the TISTR8447 after three days of nitrogen starvation.
