# Supplementary material for: Evaluation of strategies for improving the transgene expression in an oleaginous microalga Scenedesmus acutus
Source: BMC Biotechnol. 2019 Jan 10;19:4. doi: 10.1186/s12896-018-0497-z (PMC6327543; doi:10.1186/s12896-018-0497-z)
Supplement: Supplementary file 5 — Transformation rates of the TISTR8447 using four Agrobacterium strains. (PDF 39 kb) [file 12896_2018_497_MOESM5_ESM.pdf]

Additional file 5

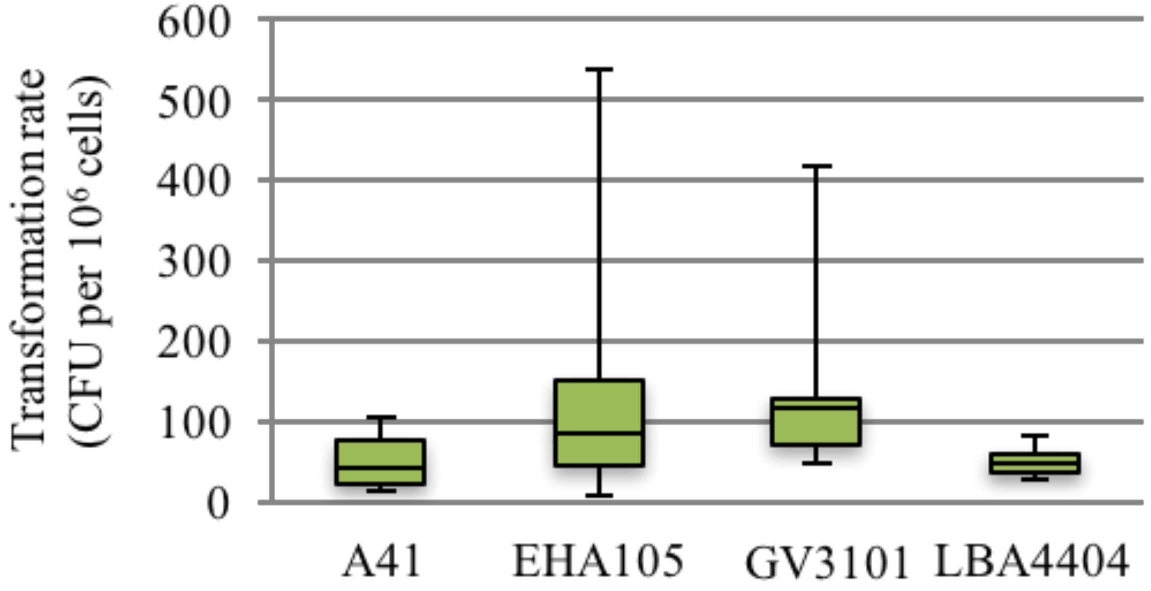

Additional file 5. Box plots represent transformation rates of the TISTR8447 using four *Agrobacterium* strains (n = 9).
