# Supplementary material for: Evaluation of strategies for improving the transgene expression in an oleaginous microalga Scenedesmus acutus
Source: BMC Biotechnol. 2019 Jan 10;19:4. doi: 10.1186/s12896-018-0497-z (PMC6327543; doi:10.1186/s12896-018-0497-z)
Supplement: Supplementary file 6 — Agrobacterium-mediated transformation of S. acutus TISTR8447. (PDF 446 kb) [file 12896_2018_497_MOESM6_ESM.pdf]

Additional file 6

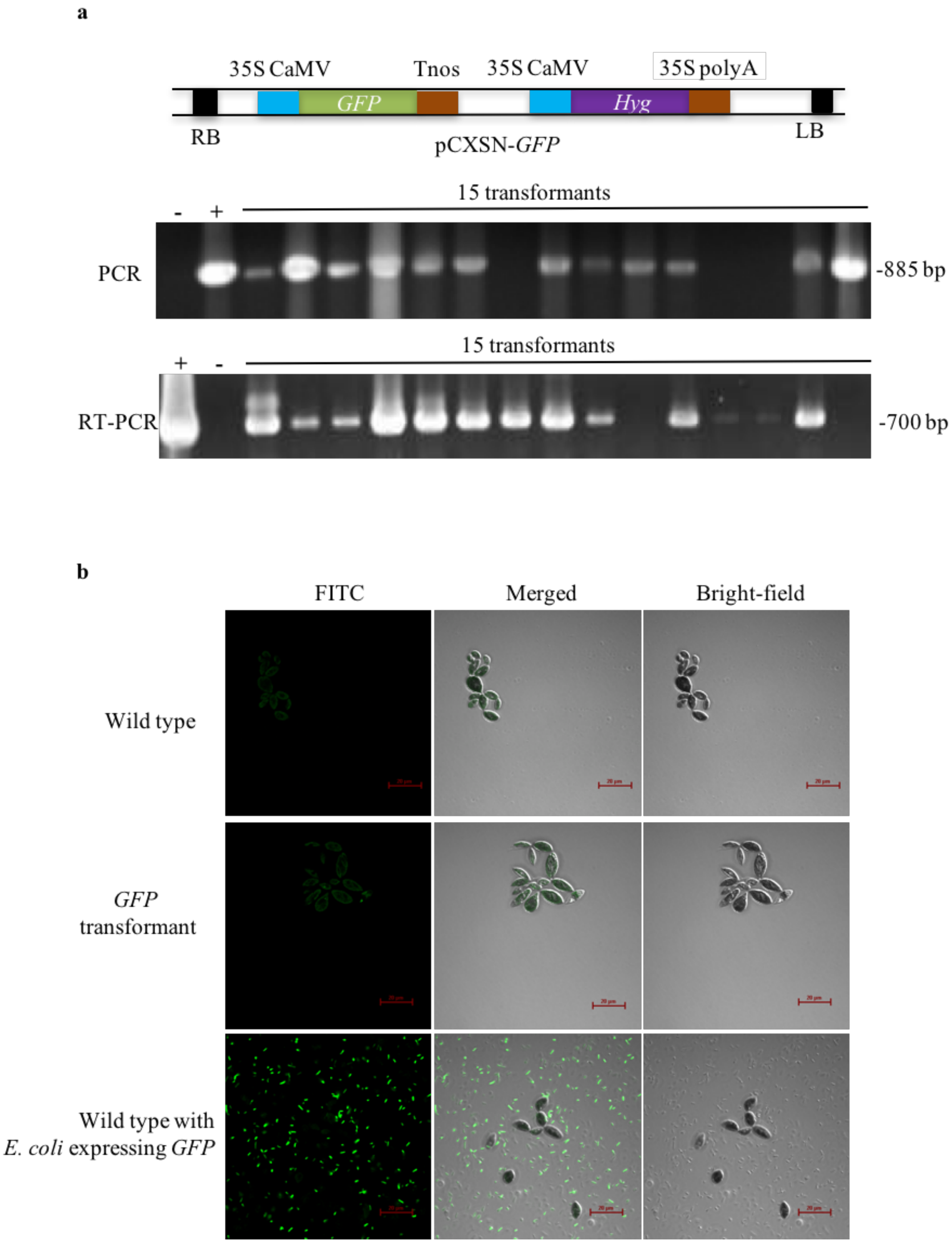

Additional file 6. *Agrobacterium*-mediated transformation of *S. acutus* TISTR8447. a. The expression construct based on pCXSN harboring *GFP* reporter. PCR analysis of 15 independent transformants for the *aphIV* transgene, and RT-PCR analysis of other 15 independent transformants for *aphIV* transcript. (-) indicates DNA or cDNA of wild type controls, and (+) indicates transformation plasmid. b. Confocal microscopy for GFP signals of TISTR8447 and *GFP* transformants. For the lowest panel, *E. coli* expressing *GFP* and TISTR8447 was mixed before the observation.
