# Supplementary material for: Evaluation of strategies for improving the transgene expression in an oleaginous microalga Scenedesmus acutus
Source: BMC Biotechnol. 2019 Jan 10;19:4. doi: 10.1186/s12896-018-0497-z (PMC6327543; doi:10.1186/s12896-018-0497-z)
Supplement: Supplementary file 7 — The schematic of pCreZ vectors. (PDF 64 kb) [file 12896_2018_497_MOESM7_ESM.pdf]

**Additional file 7**

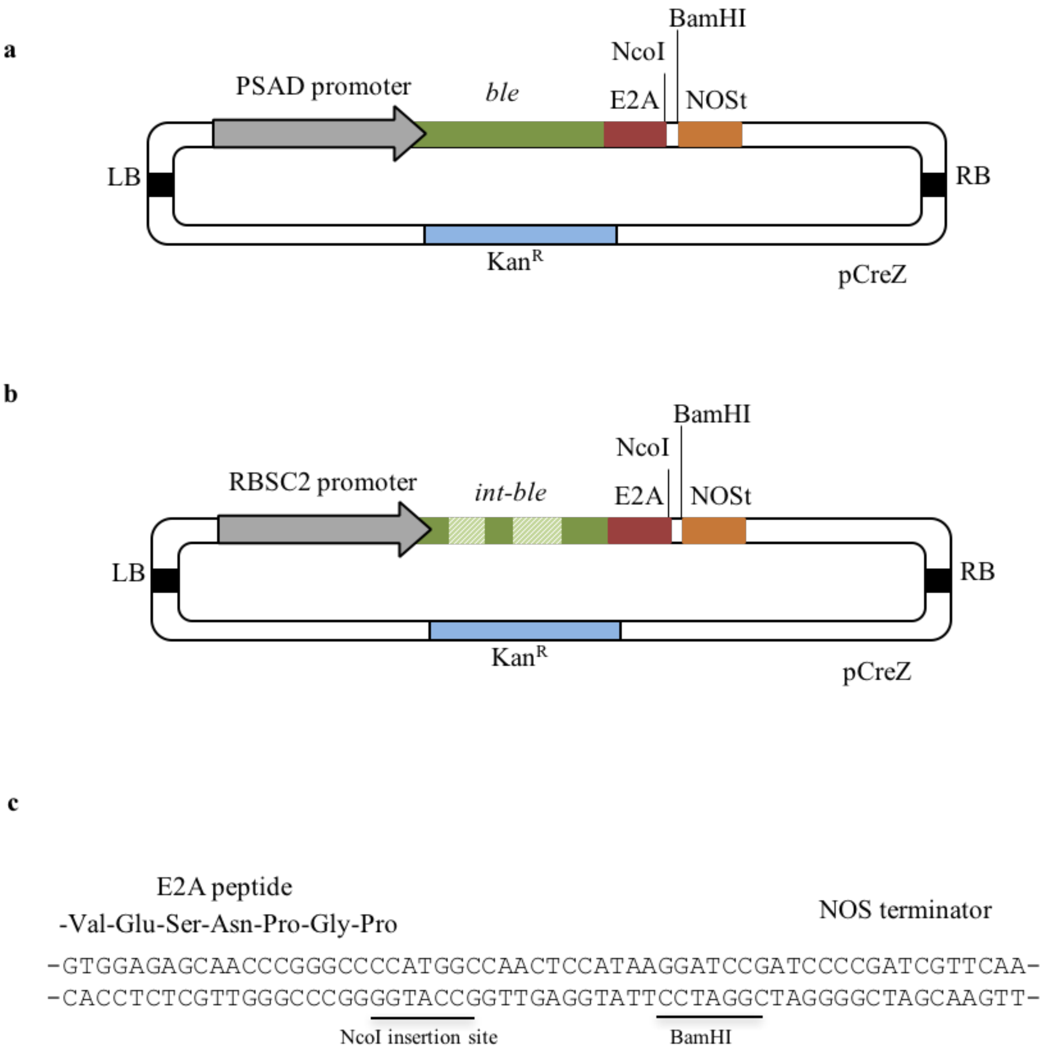


Additional file 7. The schematic of pCreZ vectors containing (b) *ble::E2A* (b)or (c) *int-ble::E2A* (*ble* with introns). (c) NcoI insertion site at the end of *E2A* sequence for in frame insertion is indicated.
