# Supplementary material for: Evaluation of strategies for improving the transgene expression in an oleaginous microalga Scenedesmus acutus
Source: BMC Biotechnol. 2019 Jan 10;19:4. doi: 10.1186/s12896-018-0497-z (PMC6327543; doi:10.1186/s12896-018-0497-z)
Supplement: Supplementary file 8 — Western analysis of CrPSY::E2A::mCherry fusion protein with extended exposure times. (PDF 127 kb) [file 12896_2018_497_MOESM8_ESM.pdf]

**Additional file 8**

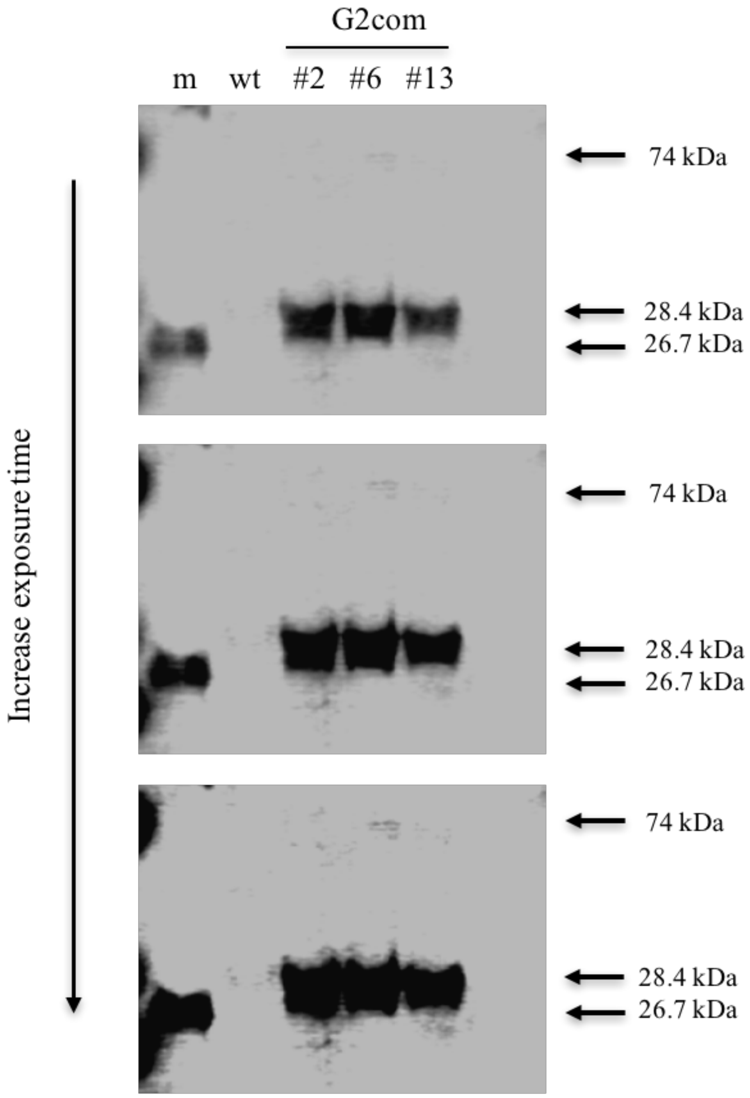


Additional file 8. Western analysis of CrPSY::E2A::mCherry fusion protein with extended exposure times. (m) indicates mCherry from *E. coli* and (wt) represents wild type TISTR8447.
