# Supplementary material for: Evaluation of strategies for improving the transgene expression in an oleaginous microalga Scenedesmus acutus
Source: BMC Biotechnol. 2019 Jan 10;19:4. doi: 10.1186/s12896-018-0497-z (PMC6327543; doi:10.1186/s12896-018-0497-z)
Supplement: Supplementary file 9 — Oligonucleotide primer sequences. (PDF 111 kb) [file 12896_2018_497_MOESM9_ESM.pdf]

Additional file 9.

Additional file 10. Oligonucleotide primer sequences.

Primers	Sequence 5'-3'
GFP-F	CACCATGGTCTCCAAGGGCGAG
GFP-R	TTACTTGTACAGCTCGTCC
pplv-F	CCAACTCCATAAGGATCCG
pplv-R	GATAACGTTACACCACAATATATC
pasd-F	GATATATTGTGGTGTAACGTTATCCTCGAGAGGGAGGCCTCACCAATCG
pasd-R	GGGATTGTGCGTCATGGTGAATTCGGCTTGTTGTGAGTAGCAG
rbcs-F	GATATATTGTGGTGTAACGTTATCCTCGAGATTTAAATGCCAGAAGGAG
Zeo-F	ACTGCTACTCACAACAAGCCGAATTCACGATGGCCAAGTTGACCAGTG
Zeo-E2A-R	GGGGCCCGGGTTGCTCTCCACGTCGCCCGCCAGCTTCAGCAGGTCGAA GTTCAGGGTCTGCTTCACCGGGGCGTCCTGCTCCTCGGCCACG
HA-mcherry-F	CGTGGAGAGCAACCCGGGCCCCATGTACCCCTACGACGTGCCCGACTA CGCTCCAATACTTATGGTGAGCAAGGGCGAGG
mcherry-R	GATCGGATCCTTATGGAGTTGGCTTACTTGTACAGCTCGTC
cry1-1-F	CTACTCACAACAAGCCGAATTCCCCATGAGACGGTCGTGAC
cry1-1-TtoC-R	GAACGATCGGGGATCGGATCCCTACAGGCGGCGACCGCGGC
crpsy-F	CTCACAACAAGCCGAATTCACGATGAACTTCAGGACCGCGC
crpsy-R	GTTCAGGGTCTGCTTCACCGGGGCCCGGCGGCGTGCGGGCATG
E2A-F	GCCCCGGTGAAGCAGACCC
pasd-crpsy-R	CGTGAATTCGGCTTGTTGTGAG
Hygro-F	GAAGAATCTCGTGCTTTCAG
Hygro-R	GGCGAGTACTTCTACACAGC
RT-Hygro-F	AATCTCGTGCTTTCAGCTTCG
RT-Hygro-R	ATATACGCCCGGAGTCGTGG
PSY seq-F	CTTCAAGCCAACGGCAG
PSY seq-R	CGCCATCATCTTCTGGTAG
