# Supplementary material for: Evaluation of strategies for improving the transgene expression in an oleaginous microalga Scenedesmus acutus
Source: BMC Biotechnol. 2019 Jan 10;19:4. doi: 10.1186/s12896-018-0497-z (PMC6327543; doi:10.1186/s12896-018-0497-z)
Supplement: Supplementary file 10 — The full length immuno blot of G2 complemented strains. (PDF 170 kb) [file 12896_2018_497_MOESM10_ESM.pdf]

Additional file 10

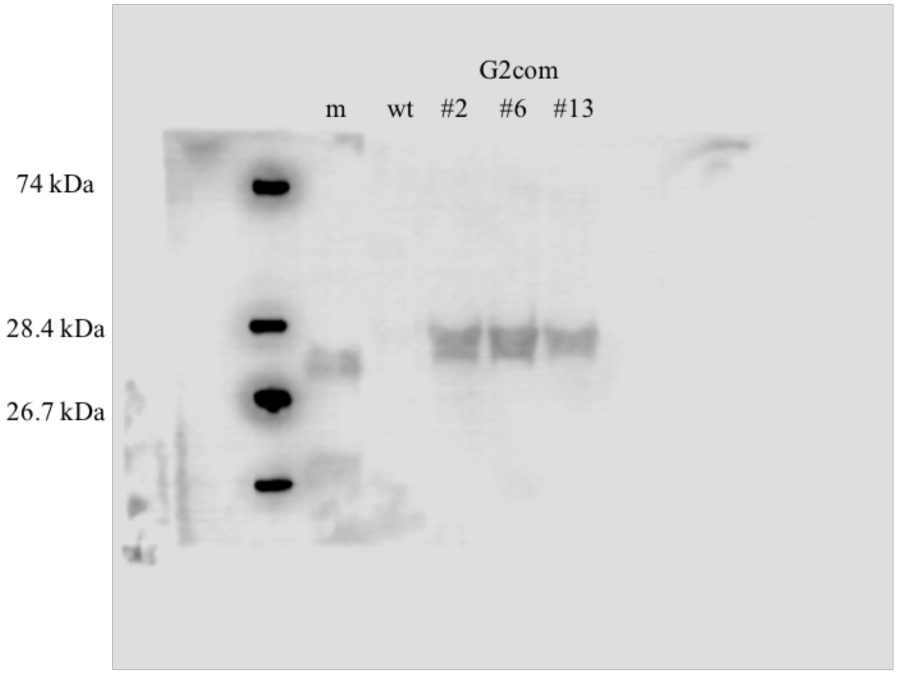

Additional file 9. The full length immuno blot of G2 complemented strains of CrPSY::E2A::mCherry fusion protein using anti-HA.
